# A Machine Learning-Based Raman Spectroscopic Assay for the Identification of *Burkholderia mallei* and Related Species

**DOI:** 10.3390/molecules24244516

**Published:** 2019-12-10

**Authors:** Amira A. Moawad, Anja Silge, Thomas Bocklitz, Katja Fischer, Petra Rösch, Uwe Roesler, Mandy C. Elschner, Jürgen Popp, Heinrich Neubauer

**Affiliations:** 1 Friedrich-Loeffler-Institut, Institute of Bacterial Infections and Zoonoses, Naumburger Str. 96a, 07743 Jena, Germany; amira.moawad@fli.de (A.A.M.); Katja.Fischer@fli.de (K.F.); heinrich.neubauer@fli.de (H.N.); 2 Animal Health Research Institute, Agricultural Research Center, 12618 Dokki-Giza, Egypt; 3 Institute of Physical Chemistry and Abbe Center of Photonics, Friedrich Schiller University, Helmholtzweg 4, 07743 Jena, Germany; anja.silge@uni-jena.de (A.S.); thomas.bocklitz@leibniz-ipht.de (T.B.); petra.roesch@uni-jena.de (P.R.); 4 InfectoGnostics Research Campus Jena, Center of Applied Research, Philosophenweg 7, 07743 Jena, Germany; 5 Leibniz-Institute of Photonic Technology, Member of the Leibniz Research Alliance – Leibniz Health Technologies, Albert-Einstein-Str. 9, 07745 Jena, Germany; 6 Institute for Animal Hygiene and Environmental Health, Free University Berlin, Robert-von Ostertag-Str. 7–13, 14163 Berlin, Germany; Uwe.Roesler@fu-berlin.de

**Keywords:** Glanders, melioidosis, Raman spectroscopy, SVM, PCA, *Burkholderia mallei*, *Burkholderia pseudomallei*, heat inactivation

## Abstract

*Burkholderia (B.) mallei*, the causative agent of glanders, and *B. pseudomallei*, the causative agent of melioidosis in humans and animals, are genetically closely related. The high infectious potential of both organisms, their serological cross-reactivity, and similar clinical symptoms in human and animals make the differentiation from each other and other *Burkholderia* species challenging. The increased resistance against many antibiotics implies the need for fast and robust identification methods. The use of Raman microspectroscopy in microbial diagnostic has the potential for rapid and reliable identification. Single bacterial cells are directly probed and a broad range of phenotypic information is recorded, which is subsequently analyzed by machine learning methods. *Burkholderia* were handled under biosafety level 1 (BSL 1) conditions after heat inactivation. The clusters of the spectral phenotypes and the diagnostic relevance of the *Burkholderia* spp. were considered for an advanced hierarchical machine learning approach. The strain panel for training involved 12 *B. mallei*, 13 *B. pseudomallei* and 11 other *Burkholderia* spp. type strains. The combination of top- and sub-level classifier identified the mallei-complex with high sensitivities (>95%). The reliable identification of unknown *B. mallei* and *B. pseudomallei* strains highlighted the robustness of the machine learning-based Raman spectroscopic assay.

## 1. Introduction

Most of the species belonging to the genus *Burkholderia* are known as plants’ associated pathogens with a soil reservoir. Two important exceptions are *B. mallei* and *B. pseudomallei*, which are implicated in life-threatening infections in human and animals. Genetic similarities and serological cross reactions between both pathovars make the identification and differentiation from each other difficult and challenging. The high infectious potential of both agents, increased resistance against many antibiotics and their small infectious dose, imply a need for fast and robust identification methods.

*B. mallei* is a Gram-negative non-motile bacterium belonging to the family *Burkholderiaceae* that mainly affects equines, causing the notifiable zoonotic disease glanders [1]. Glanders in equids is endemic in North Africa, South America, Middle East, and Asia [2]. Infection can be transmitted through direct contact with infected animals, skin cuts and abrasions, aerosol inhalation, and ingestion of contaminated drinking water and meat. The predominant generalized clinical signs are fever, drooping of the head, labored breathing, emaciation, swelling of limbs and joints. The cutaneous manifestation by multiple popular or pustular nodules and sometimes the typical yellowish-green nasal discharge with or without ulcerous nodules on the nasal mucosa can be observed [3]. The meat of infected equids can act as a reservoir for carnivorous animal infection [4]. The disease in human is occupational, affecting mainly veterinarians and laboratory and slaughterhouse workers in addition to horse owners. *B. mallei* is a host-adapted pathogen and has no environmental reservoir [5]. In contrast, its closely related species *B. pseudomallei* is a saprophyte with a reservoir in soil [6]. *B. pseudomallei* is a Gram-negative, motile, aerobic, nonspore-forming, and intracellular pathogen with a high resistance to environmental conditions [7]. The organism causes serious invasive infections in humans (including septicemia and pneumonia) and is the causative agent of melioidosis, an endemic disease affecting humans and many animal species in tropical areas with a high fatality rate [8,9]. It has frequently been reported in recent decades that melioidosis is an endemic disease of public health importance in Southeast Asia and Australia [10,11]. 

*B. mallei* and *B. pseudomallei* cause similar clinical symptoms in human and animals. The conventional microbiological identification for both have the disadvantages of being time and labor consuming and have to be performed in biosafety level 3 (BSL 3) conditions [12]. The soil bacterium *B. thailandensis* is phenotypically and genetically related to *B. mallei* and *B. pseudomallei*. Nonetheless, it shows less pathogenicity [6,13]. Since *B. thailandensis* and *B. pseudomallei* share the same reservoir, the appearance of *B. thailandensis* frequently decreases the assay specificity for the hazardous agents [13,14].

The *B. cepacia* complex (comprising the *B. cepacia, B. multivorans, B. stabilis, B. ambifaria, B. dolosa,* and *B. cenocepacia* spp.) is occupying ecological niches ranging from soil to hospital environments. Those species are considered as opportunistic pathogens to humans. They are suspected to cause cystic fibrosis [15]. *B. glathei* and *B. phytofirmans* represent the non-pathogen species of the genus *Burkholderia* [16,17,18].

The classification of *B. mallei* and *B. pseudomallei* as category B biothreat agents on one side and the increasing spread of pathogens due to international animal transport on the other side provoked researches to evaluate innovative diagnostic strategies to differentiate between both pathovars at one time. Beside the serodiagnosis, DNA microarray-based detection methods and matrix-assisted laser desorption/ionization mass spectrometry (MALDI-TOF MS) are evaluated by various research groups for the above-mentioned task [14,19,20].

The concept of Raman spectroscopic differentiation of bacteria combines the physical recording of bacterial Raman spectra with machine learning on validated reference spectra [21,22,23,24]. Stöckel et al. reported the successful differentiation between *B. mallei* and *B. pseudomallei* by Raman spectroscopy after inactivation of the bacteria with formaldehyde to handle them under BSL 1 condition [25]. Raman spectroscopy-named after Indian physicist C.V Raman-is a modern analytical tool, which uses monochromatic light sources in the visible, infrared, or ultraviolet range to investigate the biochemical composition of specimen. By simply irradiating a sample with laser light, the molecular composition of the probed sample volume can be analyzed. The photons, which are inelastically scattered by molecular bonds, are analyzed spectroscopically and the intensity of the inelastic scattering is plotted as a Raman spectrum [26]. Since Raman scattering can be observed through a microscope to measure very small sample volumes such as single bacterial cells, it became a promising tool for a wide range of microbiological applications. Within the cell’s structure, a phenotypic specific mixture of biochemical components is present. Probing a single bacterium results in a complicated Raman spectrum exhibiting overlapping Raman peaks originating from the cell’s typical components for example lipids, proteins, DNA/RNA, pigments and storage materials. Such a Raman spectrum acts as a molecular pattern, which consists of multiple features and can hardly be interpreted by comparing with a single reference spectrum. To utilize these Raman spectra for microbial diagnostic and bacterial identification, the spectral information is analyzed by multivariate statistics and machine learning. After many replicates, the class-specific Raman spectral pattern will be learned, and the algorithm can model the differences between the bacterial classes of interest [24].

Depending on the extent of distinguishable spectral phenotypes, the identification of bacteria based on Raman spectroscopic data is either successful, providing imprecise prediction or fail. It has been shown that the learning performance improves considerably if the expected biological or biochemical variances of a certain spectral phenotype are included in the training [27]. Phenotypical variations that typically mirrored in the Raman spectrum are contributed to: (*i*) The ecological setting from which the bacteria originate, (*ii*) the isolation procedures for the bacterial cells from their habitat, and (*iii*) the inactivation techniques to handle potential pathogen germs [28,29,30,31,32]. Furthermore, it was shown that the data pre-processing and a proper compilation of training collectives for the supervised machine learning in combination with hierarchical classification approaches improve the identification outcome significantly [27,33,34,35,36,37,38,39]. Once a reliable statistical model is established, the microbial diagnostic based on Raman spectroscopy is not dependent on time consuming cultivation, molecular or biochemical reactions. Only a small number of single cells (<100 isolated cells) or a minimum of biomass can uncover the identity of a specimen with a high level of accuracy. The only pre-requisite processing is the nondestructive isolation of the bacterial cells from the sample matrix to probe single intact cells [23]. The present study evaluates the reproducibility of a Raman based differentiation of *Burkholderia* spp. previously reported by Stöckel et al. carried out by an independent research laboratory and with an independent measurement set-up. In contrast to the study from Stöckel et al. bacteria in the current study are inactivated by heat instead of formaldehyde-inactivation to perform the analysis under biosafety level 1 conditions [25]. This study aims mainly to differentiate between the hazardous agents *B. mallei* and *B. pseudomallei* on a single cell level. It is investigated to which extent the spectral phenotypes form clusters analogous to the taxonomic pre-determined *Burkholderia* species. The potential to additionally detect and differentiate further relevant *Burkholderia* species is discussed. A representative panel of strains compromising *Burkholderia* from cell culture selections, round-robin tests, and well-characterized isolates (see Table 1) are measured to find out which spectral phenotypes are interfering the performance of the classification. According to the observed spectral phenotypes, classification tasks are defined to train predictive models for the stepwise differentiation of the most relevant *Burkholderia* classes. The performance of the classification is validated by independent batch cultures of the test strains. Finally, the statistic models are evaluated by the identification of *Burkholderia* strains, which are not included in the training database. 

## 2. Material and Methods

### 2.1. Micobiology

The strains listed in Table 1 were stored in cryovials containing a cryopreservative (MICROBANK, Mast Diagnostica, Reinfeld, Germany) at −80 °C until further investigation. Four batches of each strain were cultivated onto culture plates (NCBAGL) produced from nutrient agar (OXOID, Wesel, Germany) supplemented with 7.5% calf blood (Fiebig-Nährstofftechnik GbR, Idstein, Germany) and 10% glycerol (Merck, Darmstadt, Germany) for 24 h at 37 °C. Using a 1 μL inoculation loop, a part of the cultivated strains was scraped from plates, suspended in 1 mL of 0.9% NaCl solution and heated in thermomixer at 99 °C for 15 minutes under shaking at 400 rpm (MHL23, HLC BioTech, Pforzheim, Germany. The viability of the bacteria after inactivation was tested by growth control. For this 100 µL of each heat inactivated sample was dispensed on NCBAGL plates and incubated for 7 days at 37 °C. The successful inactivation could thus be proven in all samples. The prepared batches were washed 3 times using 500 μL distilled water and centrifuged at 8000× *g* (Eppendorf 5418, Eppendorf, Hamburg, Germany) for 5 minutes. Finally, 50 μL of the suspension were applied to a nickel foil, air-dried and provided to the Raman spectroscopic investigation.

### 2.2. Raman Spectroscopy

Raman spectra were collected with a Raman microscope (BioParticle Explorer, rap.ID Particle Systems GmbH, Berlin, Germany). A solid-state frequency doubled Nd:Yag module (Cobolt Samba, 25 mW, Cobolt AB, Solna, Sweden) with an excitation wavelength of 532 nm was used. The laser light was focused through an 100x objective (Olympus MPlanFLN 100xBD) onto the sample. This result in a spot size <1 µm laterally so that approximately 7 mW hit the sample.The bacteria were measured from different regions of the specimens. The Rayleigh scattering was removed by two edge filters after collecting the 180°-backscattered light, while a thermoelectrically cooled CCD camera registered the light (Andor DV401-BV). A single-stage monochromator, consisting of a 920-line/mm grating, diffracted the backscattered light so that the spectral resolution accounted for about 8 cm^−1^. The integration time per Raman spectrum (15 to 3275 cm^−1^) was 5 seconds. Approximately 50 single-bacteria Raman spectra were measured per batch (biological replicate) and collected from four separately prepared batches for further analysis. The evaluation of the classification models was performed by using of about 20 spectra from other isolates (Table 2).

### 2.3. Data Pre-Processing 

**Figure 1:**
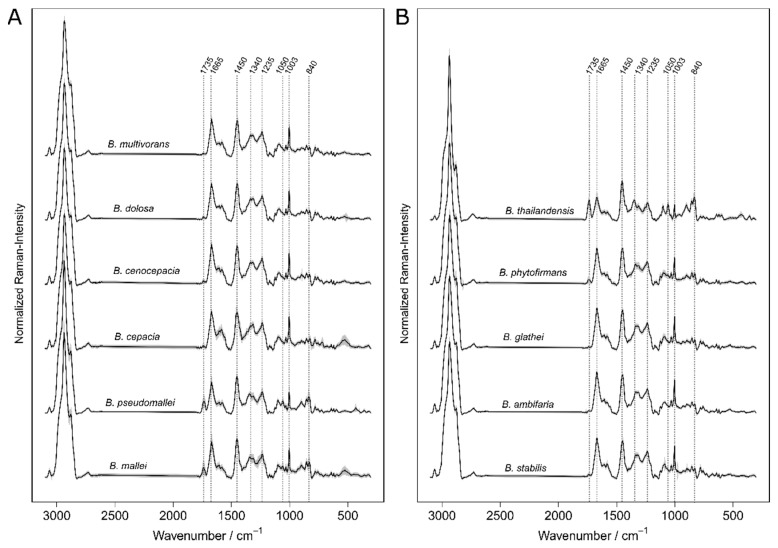
The mean spectra of the *Burkholderia* species were shown in panel **A** and **B** The grey zone around the solid lines visualizes the standard deviation of the specie’s mean. Representative Raman signals are marked.

The open source software Gnu R (version R-3.6.0 Vienna, Austria) were used for all computations [40]. The pre-processing was carried out according to principles investigated in Bocklitz et al. [41]. The cosmic spike removal was performed according to the reference [42] and the threshold was set to 10. The used wavenumber positions of the wavenumber standard (4-acetamidophenol) were 329.2, 390.9, 465.1, 504, 651.6, 710.8, 797.2, 857.9, 968.7, 1105.5, 1236.8, 1278.5, 1323.9, 1371.5, 1561.5, 1648.4, 2931.1, 3064.6, and 3102.4 cm^−1^. A polynomial of degree 3 was utilized for wavenumber calibration [43] and the background was subsequently corrected by the SNIP algorithm [44]. The used wavenumber area was 300 cm^−1^ to 3100 cm^−1^ and the wavenumber area between 1800 cm^−1^ and 2600 cm^−1^ was excluded (Figure 1). As normalization a vector normalization was applied. Spectra of burned bacteria or material artefacts were excluded from the respective data set. 

**Figure 2:**
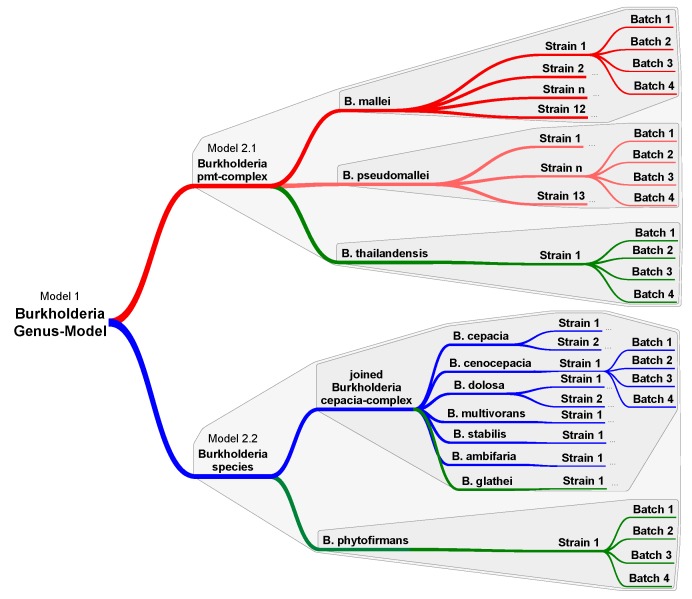
Hierarchically organized classification workflow for Burkholderia’s Raman data. Model 1 includes all Raman data and separate the p–ma–thai-complex (includes *B. mallei*, *B. pseudomallei,* and *B. thailandensis*) from other *Burkholderia* species. The dataset is split at the following level. Model 2.1 includes the Raman data of the p–ma–thai-complex and differentiates between the three species. Model 2.2 classifies the data of c-gla-phy-complex and differentiates between the cluster of *B. cepacia*-complex and non-pathogen *Burkholderia* species *B. phytofirmans*. Each Burkholderia species was represented by at least one strain and from each strain 4 batches were measured to provide biological and technical independent data sets for validation.

The number of spectra of each single batch (overview Figure 2) was reduced from the original number of recorded spectra to 20 representative spectra. A random sampling without replacement was performed (user-independent), which selects 3 spectra and merge them to a mean spectrum. This is done until no single spectrum of the data set is left (depending on the total number of spectra per batch, the last group of selected spectra may contain only two single cell spectra). After preprocessing the data were introduced to a Principal Component Analysis (PCA). A PCA score plot visualize the data’s inherent clusters without prior knowledge about the categorical (species) label. A PCA score plot of the first principal components was plotted ones for the full data set (including all *Burkholderia* species) and in the following for each determined subset (p–ma–thai-complex and c-gla-phy-complex). For visualization the mean score for each species was calculated and the standard deviation ellipse was plotted for an overview of the group extend.

### 2.4. Statistical Learning

A support vector machine (SVM was applied for machine learning in combination PCA. For each classification task an appropriate number of PCs (derived from the PCA objects of the particular classification levels) were introduced. The first 45 PCs were utilized to train the SVM of model 1, which separates the data of the *B. mallei* complex from the other *Burkholderia* species. The unbalanced class sizes were considered by a class weighted approach. The SVM model 2.1 was established by utilizing the first 40 PCs and SVM model 2.2 by using the first 25 PCs. Each SVM model was validated by a leave-one-batch-out cross-validation (LOBOCV) [27]. For this purpose, the Raman data except the data of the batches with the serial number x were used to construct a SVM model. This model was utilized to predict the classes of the left-out batch series. For example, at the top level, all 36 Burkholderia strains were included (see Figure 2). Each single batch included 20 spectra. Therefore, each hold out batch series included 1 batch × 36 strains × 20 spectra = 720 spectra and the training data 3 batches × 36 strains × 20 spectra = 2160 spectra. Accordingly model 2.1 included 26 strains and model 2.2 10 strains. This method is repeated so that spectra of each batch series are predicted once. The number of spectra per batch series used for validation purpose should not be confused with the class size. Raman data of the *Burkholderia* strains from the evaluation set (Table 2) were preprocessed separately and rotated into the respective PCA space of model 1, 2.1, and 2.2 before they were predicted by the respective SVM model. 

## 3. Results 

### 3.1. Data Management

The results of the Raman measurements are summarized in Figure 1. For each *Burkholderia* species a mean spectrum is shown. The standard deviation is drawn as the grey zone around each spectrum to visualize the within species variation. Noticeable are the spectra of *B. mallei, B. pseudomallei, B. phytofirmans,* and *B. thailandensis*. They exhibit the combined appearance of signals around 843, 1050, 1450, and 1735 cm^−1^ which are typical for Polyhydroxybutyrat (PHB), an intracellular storage material [45]. The signals of PHB were observed with different extents in the single cell Raman spectra of nearly every *Burkholderia* species (Figure S1). Overall irregularities like the appearance of PHB were equalized to some extend by summarizing the data of single cells within one culture batch to subsets of merged spectra by a randomized procedure described in the section material and methods (also visualized in the supporting information Figure S2). The reduced data matrices were used for an optimized training of the classifier. Such a data management makes the introduced approach reproducible and more robust against biological variations.

### 3.2. Classification Workflow for Burkholderia’s Raman Data

A prerequisite for machine learning and statistic modeling on Raman spectral data is a database containing validated reference spectra of bacterial cells exhibiting phenotypic variations. Different number of strains was available during the study for the representation of the *Burkholderia* species. The target species *B. mallei* and *B. pseudomallei* were available with a representative number of strains. For the remaining species one to two strains were included into the training (Table 1). For each strain, four independently cultivated batches were measured to include the variances from different culture plates and from day to day. The workflow for the data collection is visualized in Figure 2.

A task-oriented and hierarchically organized classification workflow for Burkholderia’s Raman data previously described by Stöckel et.al [25] was developed further in the present study. As a first step a PCA was applied to examine if the data contains inherent clusters. The PCA as an unsupervised data analysis tool finds the main axes of variance within a data set without prior knowledge about the categorical label. The PCA reduces the dimensionality of the Raman data by calculating a new set of principal components (PCs) to minimize redundant information without loss of spectral information.

**Figure 3:**
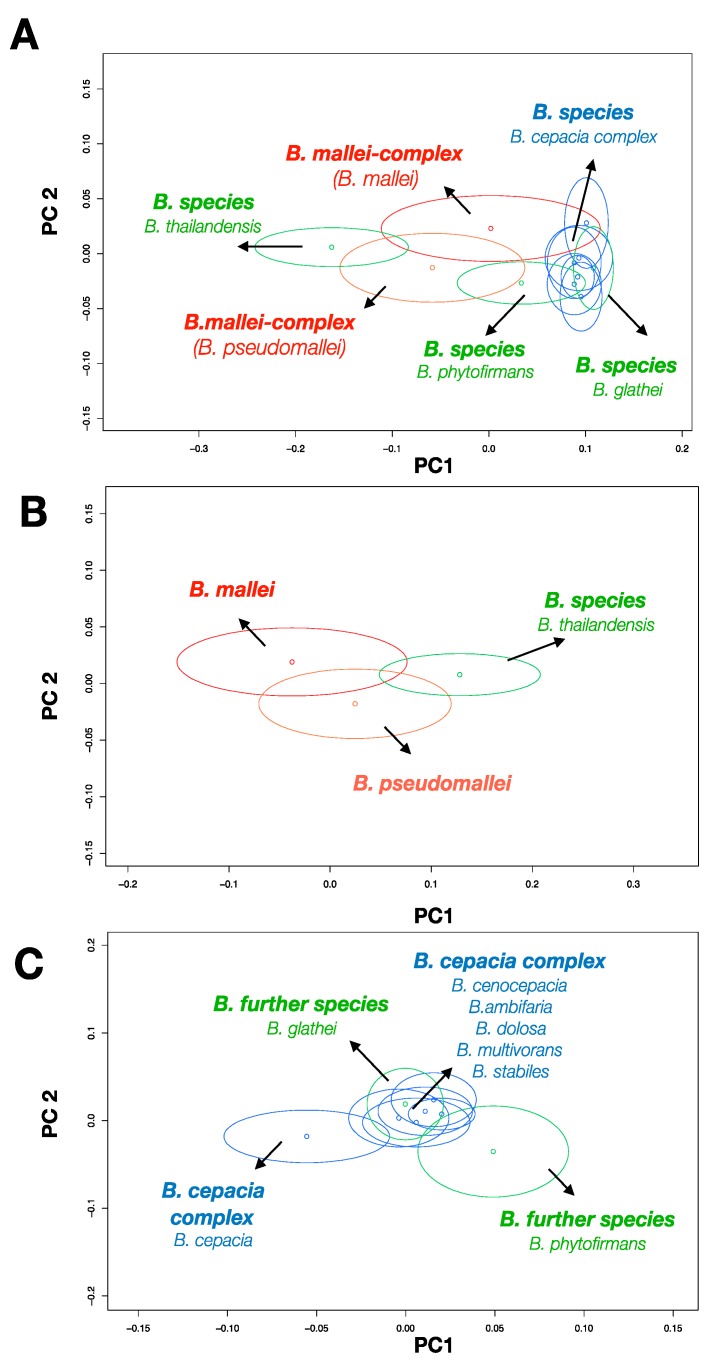
The PCA score plot shows the two directions of largest variance in the data and provides a valuable insight into the nature of Burkholderia’s Raman data. The spread of the data points of each *Burkholderia* species was visualized by the standard deviation ellipse for an overview. The colors codes the Burkholderia complexes. The obligate pathogen species of the *B. mallei*-complex are highlighted in red. The facultative pathogen species of the *B. cepacia-*complex are shown in blue and the non-pathogen *B. phytofirmans, B. glathei, and B. thailandensis* are visualized in green. **A**: The panel shows the whole *Burkholderia* data set (Model 1). **B**: Score plot of the p–ma–thai complex show three clusters (Model 2.1). **C**: Score plot of the remaining *Burkholderia* species (Model 2.2).

The PCA score plot in Figure 3A shows the two directions of largest variance in the data and provides a valuable insight into the nature of Burkholderia’s Raman data. Literally, data with similar spectral phenotypes cluster together. The spread of the data points of each Burkholderia species was visualized by one times the standard deviation ellipse as an overview. The colors code the different complexes of the genus *Burkholderia* [6,15,46]. The obligate pathogen species of the *B. mallei*-complex are highlighted in red. The facultative pathogen species of the *B. cepacia-complex* are shown in blue and the non-pathogen *B. phytofirmans, B. glathei,* and *B. thailandensis* are represented by green. The PCA score plot shows that each species overlaps with other species, which was expected for bacteria of one genus. 

Due to the clusters shown in the PCA plot, classes with shared spectral characteristics are grouped together under consideration of their diagnostic relevance. *B. pseudomallei, B. mallei, and B. thailandensis* were pooled and labelled as p–ma–thai-complex [15]. The species of the *B. cepacia-*complex pooled together with *B. glathei* and *B. phytofirmans* and joined to the ce–gla–phy-complex. Consequently, the first classification task was to differentiate between the major spectral classes of the dataset. In a next step the sub classes for the supervised machine learning were compiled. The PCA-score plot in Figure 3B show that the data of the p–ma–thai-complex fall into three clusters of the particular species. Therefore, the model 2.1 differentiates between the three species of the p–ma–thai-complex. In Figure 3C the PCA-score plots of the second data-subset are shown. The species of the *B. cepacia* complex cluster with *B. glathei* and the cluster of *B. phytofirmans* appears apart. Data of the *B. cepacia*-complex and *B. glathei* were pooled to a new joined class. Model 2.2 was trained to separate the joined *cepacia* complex from *B. phytofirmans*. Since the differentiation between the species of the *B. cepacia*-complex and *B. glathei* was not of interest, the separation of the species of the *B. cepacia-*complex was not further carried out. In summary, sub-classes are grouped following a narrowing path on classification. For each level a specific classification model was trained based on the data of the joined classes. A given decision on one level leads down to different classification paths.

The SVM is an efficient machine learning algorithm for Raman spectral data in combination with a dimensionality reduction technique like PCA. PCA reduces the number of features by choosing the most important ones that still represent a maximal part of the entire dataset. The cumulative explained variance of the principal components (PCs) for the top- and sub level data sets were examined and shown in the supporting information (Figures S3–S5). Less than 200 PCs of the 627 dimensions explain 90% of the data’s variance (the 627 dimensions derived from the 627 wavenumbers per spectrum). Within these 200 PCs the best number was screened for SVM input and adjusted depending on the complexity of the classification task. To prevent overfitting, the performance of each PCA-SVM model was optimized and tested by applying a leave one batch out cross validation (LOBOCV). This procedure provides biological and technical independent data sets for validation to get an idea of the model’s reliability. Model 1 gave the highest validation accuracy by introducing the first 45 PCs (the data set at the top level contained 2880 spectra). The complexity of the classification tasks decreased with each level in a hierarchically organized classification approach. The p–ma–thai-complex included 2000 spectra. Here, the classification result was best when 40 PCs were introduced. The ce–gla–phy-data set comprises the smallest number of strains and contained 880 spectra. Here 25 PCs were sufficient for classification.

### 3.3. Hierarchical Classification 

The confusion table of model 1 (Table 3) shows that 95.5% of the p–ma–thai-complex and 83.4% of the ce–gla–phy-complex were predicted correctly. The result reveal that model 1 is sensitive for the recognition of the p–ma–thai-complex with a considerably low false negative rate of 4.5%. The false positive rate of the p–ma–thai-complex examined by the LOBOCV was 16.6%. That means that more spectra of the ce–gla–phy-complex were misclassified by the model and therefore the specificity for the targets of the p–ma–thai-complex diminish. *B. phytofirmans* could be identified as an important interfering species because its spectral phenotype shows a strong overlap with *B. pseudomallei* (Figure 2A). 

The confusion table of model 2.1 (Table 4) shows the result for differentiation between the three species of the p–ma–thai-complex. Sensitivities of 91.4% and 91.9% for *B. mallei* and *pseudomallei* were reached respectively. For *B. thailandensis* 65% spectra were correctly classified. Misclassifications of *B. thailandensis* are mainly due to the confusion with *B. mallei* but not vice versa. In contrast, the confusion of *B. thailandensis* with *B. pseudomallei* was insignificant. 

The confusion table of model 2.2 (Table 5) summarizes the differentiation between data of the joined *B. cepacia-B. glathei* class (99.2% sensitivity) and *B. phytofirmans* (91.2% sensitivity). 

### 3.4. Identification of New Strains

**Figure 4:**
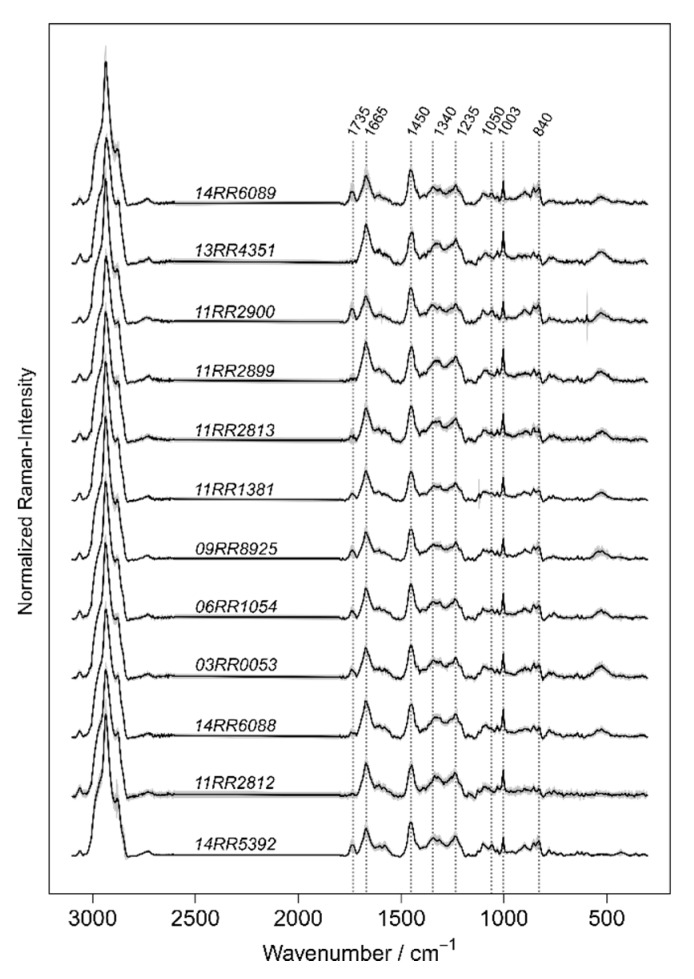
Mean spectra of the *Burkholderia* strains introduced to the hierarchical classification model for identification. The grey zone around the solid lines visualizes the standard deviation of the strain’s mean spectra.

After training and validating the classification performance of the models, their generalizability was evaluated. Raman data of new *B. mallei* and *B. pseudomallei* strains were recorded, which were not taken into account for the training. The pre-processing and the exclusion of artefacts were performed in the same way like for the training data. The only exception was the averaging procedure, which was only performed for an optimized training approach. For an overview the spectra of the test strains are plotted in Figure 4. The new data were predicted by model 1 and model 2.1 and the results presented in Table 6 and Table 7. 

According to the predictions performed by model 1 the majority of all spectra were put correctly into the joined class of the p–ma–thai-complex (Table 6). Ten of the twelve strains were properly identified with sensitivities ranging between 94% and 100%. A higher number of spectra were recorded for the strain 14RR5392, an isolate originated from a Green Iguana in Prague [47]. Four independent biological replicates were tested here and 95% of the data were correctly predicted by model 1. The lowest sensitivities achieved by model 1 ranged between 80% and 90% (strain 06RR1054 with 88.9% and 11RR2812 with 83.3% correctly identified spectra). Raman data which were assigned to the p–ma–thai complex by model 1, were introduced to model 2.1 for species determination. The results are shown in Table 7. It is noteworthy that none of spectra was misclassified as *B. thailandensis*. Misclassifications occur only infrequently between *B. mallei* and *B. pseudomallei.* For ten of the twelve strains the sensitivities ranged between 90% and 100%. The lowest level of identification accuracy was found for the *B. mallei* strain 10RR1381 with 87% accuracy. However, one should note that the classification outcome refers to the classification of single cells representing only a small percentage of the biomass of one culture or batch. The results reveal the strength of Raman spectroscopic based identification that only a small number of representatives (or a minimum of biomass) already reveal the identity of the specimen with a high level of accuracy.

## 4. Discussion

The concept of Raman spectroscopic differentiation of bacteria combines the physical recording of bacterial Raman spectra with supervised machine learning on validated reference spectra. Stöckel et al. reported that a single SVM was not capable of discriminating the Raman data of *B. mallei* and *B. pseudomallei* alongside other *Burkholderia* and *Pseudomonas* species [25]. Therefore, the Raman spectra of *B. mallei* and *B. pseudomallei* were pooled together and treated as a joined class. A top-level SVM separated this joined class from the remaining species and a sub-level SVM exclusively performs on data of *B. mallei* and *B. pseudomallei* for definitive species separation. Identification accuracies of more than 90% could be achieved on the spectra level. The hierarchically organized classification workflow for Raman data of *Burkholderia* previously described by Stöckel et.al [25] was further developed in the present study.

For an optimal supervision of the learning process, data’s inherent clusters were considered for the compilation of joined classes. Valuable insights into the nature of the Burkholderia’s Raman data are provided and information about the interfering species are delivered. By applying a LOBOCV, a realistic estimation of the strength and limits of *Burkholderia* species identification based on Raman spectroscopic data can be elucidated. The strength of the method is exhibited by the high sensitivities for the identification of the target species *B. mallei* and *B. pseudomallei* which follows the results of Stöckel et al. This is supported by the identification of the new and not referenced *B. mallei* and *B. pseudomallei* strains. The sensitives for the identification of the new *Burkholderia* strains reached for ten of the twelve strains between 90% and 100%. The results of the present study provided independent evidence of reproducibility for the classification and identification of *B. mallei* and *B. pseudomllei* based on Raman data. However, the quality of a classifier is also dependent on the specificity for the identification of a target species. The in comparison more frequently occurring misclassification of the ce–gla–phy-complex as *B. mallei* related agents at the top-level limits the reliability of the model. It is suggested that the misclassified class might be not sufficiently represented in the training data set and therefore model 1 insufficiently captures the underlying pattern of the data. This also applies to *B. thailandensis* which was represented by only one strain at the level of model 2.1. An increased classification accuracy is expected by a proper representation of the species-specific spectral phenotype. Even it is essential to have *B.thailandensis* in the database, it is important to mention that the bacterium is not an expected contaminant in sample material for clinical diagnostics, and any misclassifications could be not over-interpreted. 

In contrast, model 2.1 perfectly predicts the classes for *B. mallei* and *B. pseudomallei* in the training and generalizes to the new strains that hasn’t been utilized before. 

To address a specific diagnostic, the problem of sample size planning and the compilation of training data for machine learning has to be optimized so that the differences between species with a similar spectral phenotype can be properly modelled. The present study highlighted the potential of a machine learning-based Raman spectroscopic assay as a microbial diagnostic tool. It was shown that the performance of the method for the identification of *B. mallei* and *B. pseudomallei* could be reproduced by an independent laboratory and with independent measurement equipment. 

As a next step, the model transferability for bacterial Raman data has to be optimized. With a view to multicenter identification purposes established database can then be shared between different laboratories. Once the access to a comprehensive bacterial Raman data collection is provided, task-specific compilations of data subsets can be used to answer new upcoming diagnostic questions. 

## Figures and Tables

**Table 1:** *Burkholderia* strains used for the training data set, their origin and number of Raman spectra.

Species	Laboratory Number	Name of Strain	Source	no. Spectra
*B. mallei*	211101RR0419	Bogor	BfR	261
	300102RR0118	ATCC 23344 _Typst._	BfR	235
	061102RR0551	Bfr 237	BfR	229
	290103RR0041	Mukteswar	BfR	217
	080304RR0090	Zagreb	BfR	250
	041206RR1051	NCTC 10260	IMB	236
	041206RR1052	NCTC 10230	IMB	260
	041206RR1055	NCTC 120-Lister	IMB	266
	041206RR1056	NCTC 10247	IMB	220
	041206RR1057	BfR M2	IMB	279
	240609RR5318	Dubai7	IMB	243
	010411RR2811	Bahrain1	FLI	257
*B. pseudomallei*	041206RR1058	Holland	IMB	304
	041206RR1059	PITT 521	IMB	315
	041206RR1060	PITT 225A	IMB	315
	041206RR1061	Heckeshorn	IMB	313
	041206RR1062	NCTC 1688	IMB	297
	041206RR1063	EF15660	IMB	314
	041206RR1064	PITT 5691	IMB	272
	060406RR0740	03-04450	IMB	317
	060406RR0745	03-04448	IMB	324
	290103RR0046	ATCC 23343	BfR	315
	120107RR0019	Bozen	MSB	316
	250413RR3267	A101-10	RKI	314
	081210RR1369	Bp 9/H05410-0490	RKI	333
*B. thailandensis*	090804RR0288	DSM 13276	DSMZ	250
*B. cepacia*	130303RR0117	ATCC 25608	DSMZ	271
	120707RR0672	DSM 7288	DSMZ	326
*B. cenocepacia*	180507RR0377	ATCC BAA-245	DSMZ	295
*B. dolosa*	180507RR0376	DSM 16088	DSMZ	331
	030718RR17093	DSM 26124	DSMZ	365
*B. multivorans*	180507RR0375	DSM 13243	DSMZ	337
*B. stabilis*	180507RR0378	DSM 16586	DSMZ	318
*B. ambifaria*	150408RR2192	DSM 16087	DSMZ	331
*B. glathei*	150408RR2194	ATCC 29195	DSMZ	294
*B. phytofirmans*	111109RR8565	DSM 17436	DSMZ	332

**DSMZ**: German Collection of Microorganisms and Cell Cultures, Braunschweig; **BfR**: Federal Institute for Risk Assessment, Berlin; **IMB:** Sanitary Academy of the Armed Forces, Munich; **RKI:** Project QUANDHIP, Robert Koch Institute, Berlin; **FLI:** Friedrich-Loeffler-Institut, Jena, **MSB**: Medical Service Bozen.

**Table 2:** *Burkholderia* strains used for validation, their origin and number of Raman spectra.

Species	Laboratory Number	Name of Strain	Source	no. Spectra
*B. mallei*	040203RR0053	M1	BfR	28
	041206RR1054	ATCC 23344_Typst._	IMB	36
	251109RR8925	ATCC 23344_Typst._	RKI	21
	081210RR1381	ATCC 23344 _Typst._	RKI	23
	010411RR2812	010411RR2812	FLI	42
	010411RR2813	010411RR2813	FLI	26
	040411RR2899	040411RR2899	FLI	30
	040411RR2900	040411RR2900	FLI	40
	100713RR4351	NCTC 10245	RKI	25
	150614RR6088	M3	BfR	68
	150614RR6089	U5	BfR	24
*B. pseudomallei*	120214RR5392	VB976100	VML	302

**DSMZ**: German Collection of Microorganisms and Cell Cultures, Braunschweig; **BfR**: Federal Institute for Risk Assessment, Berlin; **IMB**: Sanitary Academy of the Armed Forces, Munich; **RKI**: Project QUANDHIP, Robert Koch Institute, Berlin; **FLI**: Friedrich-Loeffler-Institut, Jena **VML**: Vet. Med. Laboratory GmbH, Ludwigsburg; **MSB**: Medical Service Bozen.

**Table 3:** Results of the *Leave one Batch Out* cross-validation of Model 1.

		True	
**Model 1**		p–ma–thai-complex ^1^	c-gla-phy-complex ^2^
**Identified as**	p–ma–thai-complex ^1^	1986	133
	c-gla-phy-complex ^2^	94	667
Sensitivities in %		95.5	83.4

^1^ Burkholderia p–ma–thai-complex (includes B. mallei, B. pseudomallei and B. thailandensis). ^2^ Burkholderia c-gla-phy-complex (includes B. cepacia-complex, B. glathei, B. phytofirmans).

**Table 4:** Results of the *Leave one Batch Out* cross-validation of Model 2.1.

		True		
**Model 2.1**		*B. mallei*	*B. pseudomallei*	*B. thailandensis*
**Identified as**	*B. mallei*	877	80	23
	*B. pseudomallei*	54	956	5
	*B.thailandensis*	29	4	52
Sensitivities in %		91.4	91.9	65

**Table 5:** Results of the *Leave one Batch Out* cross-validation of Model 2.2.

		True	
**Model 2.2**		joined cepacia complex ^3^	*B. phytofirmans*
**Identified as**	joined cepacia complex ^3^	714	7
	*B. phytofirmans*	6	73
Sensitivities in %		99.2	91.3

^3^*Burkholderia* joined cepacia complex (includes species of the B. *cepacian complex* and *B. glathei*).

**Table 6:** Results of the identification of unknown *Burkholderia* strains. Results of model 1 summarized as confusion-table of the strain label versus the predicted classes.

		True											
Model 1		03RR0053	06RR1054	09RR8925	10RR1381	11RR2812	11RR2813	11RR2899	11RR2900	13RR4351	14RR5392	14RR6088	14RR6089
**Identified as**	p–ma–thai-complex ^1^	28	32	20	23	35	25	29	40	25	287	64	24
	c-gla-phy-complex ^2^	0	4	1	0	7	1	1	0	0	15	4	0
Sensitivities in %		100	88.9	95.2	100	83.3	95.2	96.7	100	100	95	94.1	100

^1^ Burkholderia p–ma–thai-complex (includes B. mallei, B. pseudomallei, and B. thailandensis). ^2^ Burkholderia c-gla-phy-complex (includes B. cepacia-complex, B. glathei, B. phytofirmans).

**Table 7:** Data identified by model 1 as p–ma–thai-complex were introduced to model 2.1 for species identification.

		True											
Model 2.1		03RR0053	06RR1054	09RR8925	10RR1381	11RR2812	11RR2813	11RR2899	11RR2900	13RR4351	14RR5392	14RR6088	14RR6089
**Identified as**	*B.mallei*	28	32	20	20	31	23	29	40	24	24	63	24
	*B. pseudomallei*	0	0	0	3	4	2	0	0	1	263	1	0
	*B. thailandensis*	0	0	0	0	0	0	0	0	0	0	0	0
Sensitivities in %		100	100	100	87	88.6	92	100	100	96	91.6	98.4	100

## Supplementary Materials

The following are available online at https://www.mdpi.com/1420-3049/24/24/4516/s1.
