# Supplementary material for: A Machine Learning-Based Raman Spectroscopic Assay for the Identification of Burkholderia mallei and Related Species
Source: Molecules. 2019 Dec 10;24(24):4516. doi: 10.3390/molecules24244516 (PMC6943587; doi:10.3390/molecules24244516)
Supplement: Supplementary file 1 [file molecules-24-04516-s001.pdf]

Supporting Information

A machine learning-based Raman spectroscopic assay for the identification of *Burkholderia mallei* and related species

Amira A. Moawad[1]†, Anja Silge[3,4]†, Uwe Roesler[2], Katja Fischer[1], Petra Rösch[3]
Thomas Bocklitz[4,5], Mandy C. Elschner[1*], Jürgen Popp[3,4,5*] and Heinrich Neubauer[1]

[1]Friedrich-Loeffler-Institute, Institute of Bacterial Infections and Zoonoses, Naumburger Str. 96a, 07743 Jena, Germany

[2]Institute for Animal Hygiene and Environmental Health, Free University Berlin, Robert-von Ostertag-Str.
7-13, 14163 Berlin, Germany.

[3]Institute of Physical Chemistry and Abbe Center of Photonics, Friedrich Schiller University, Helmholtzweg 4, Jena, Germany

[4]InfectoGnostics Research Campus Jena, Center of Applied Research, Philosophenweg 7, 07743 Jena, Germany

[5]Leibniz-Institute of Photonic Technology, Member of the Leibniz Research Alliance – Leibniz Health Technologies, Albert-Einstein-Str. 9, 07745 Jena, Germany

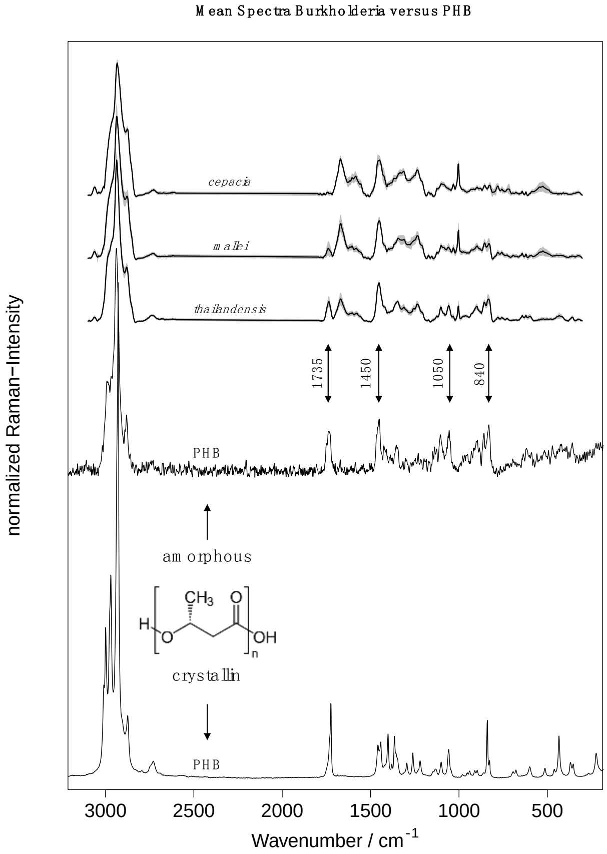

Fig. S1 Reference Spectra of crysatallin and amorphous PHB and *Burkholderia* species with varying PHB content.

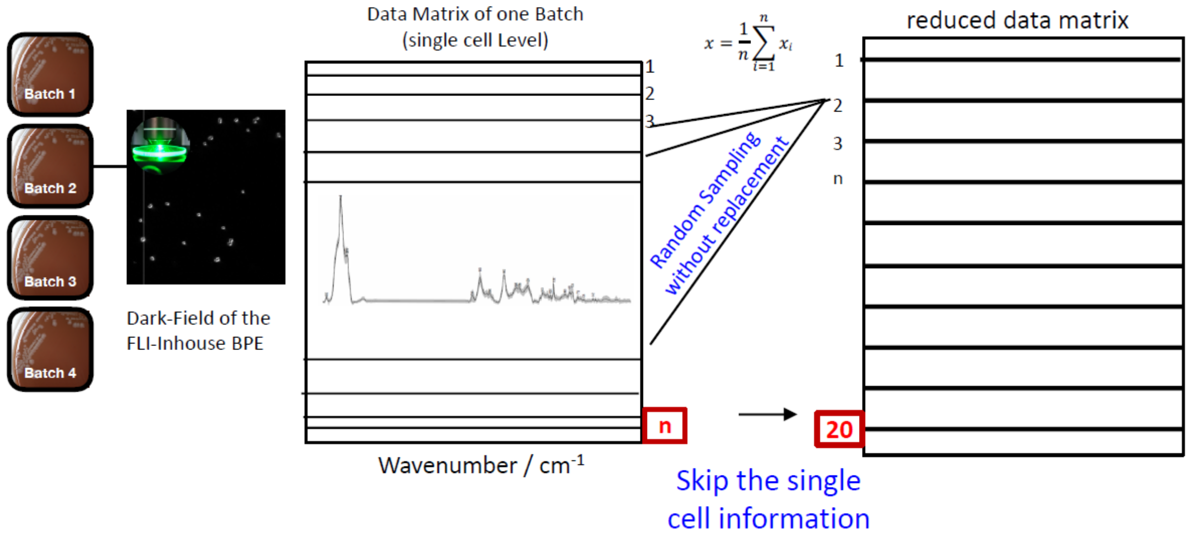

Fig.S2 Datamanagement for the Training data. Calculating of the reduced data matrix for the training of the classification models.

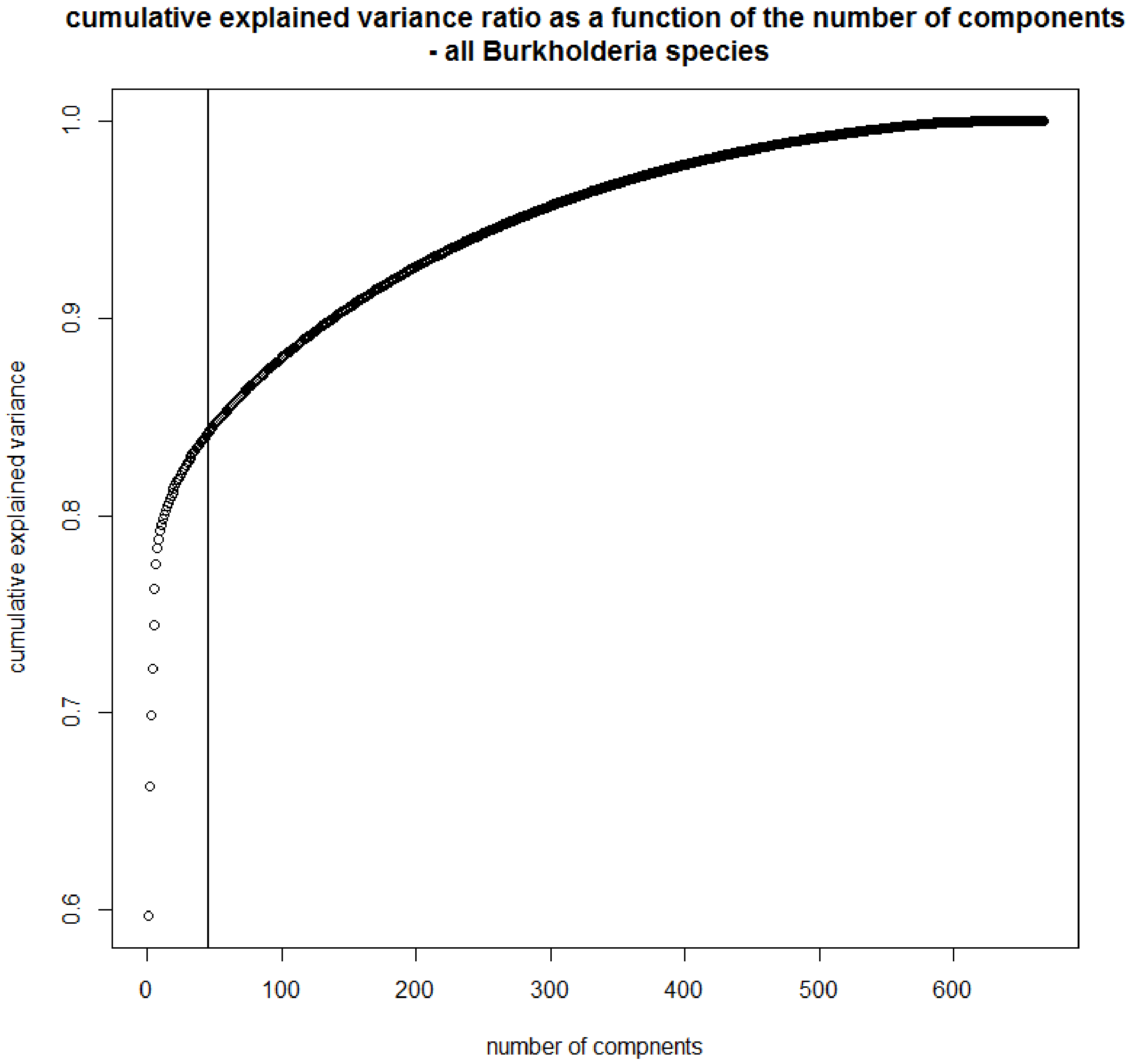

Fig. S3: The cumulative explained variance of the principal components (PCs) for the top- level data set (model 1). Model 1 gave the highest validation accuracy by introducing the first 45 PCs, indicated by the solid line.

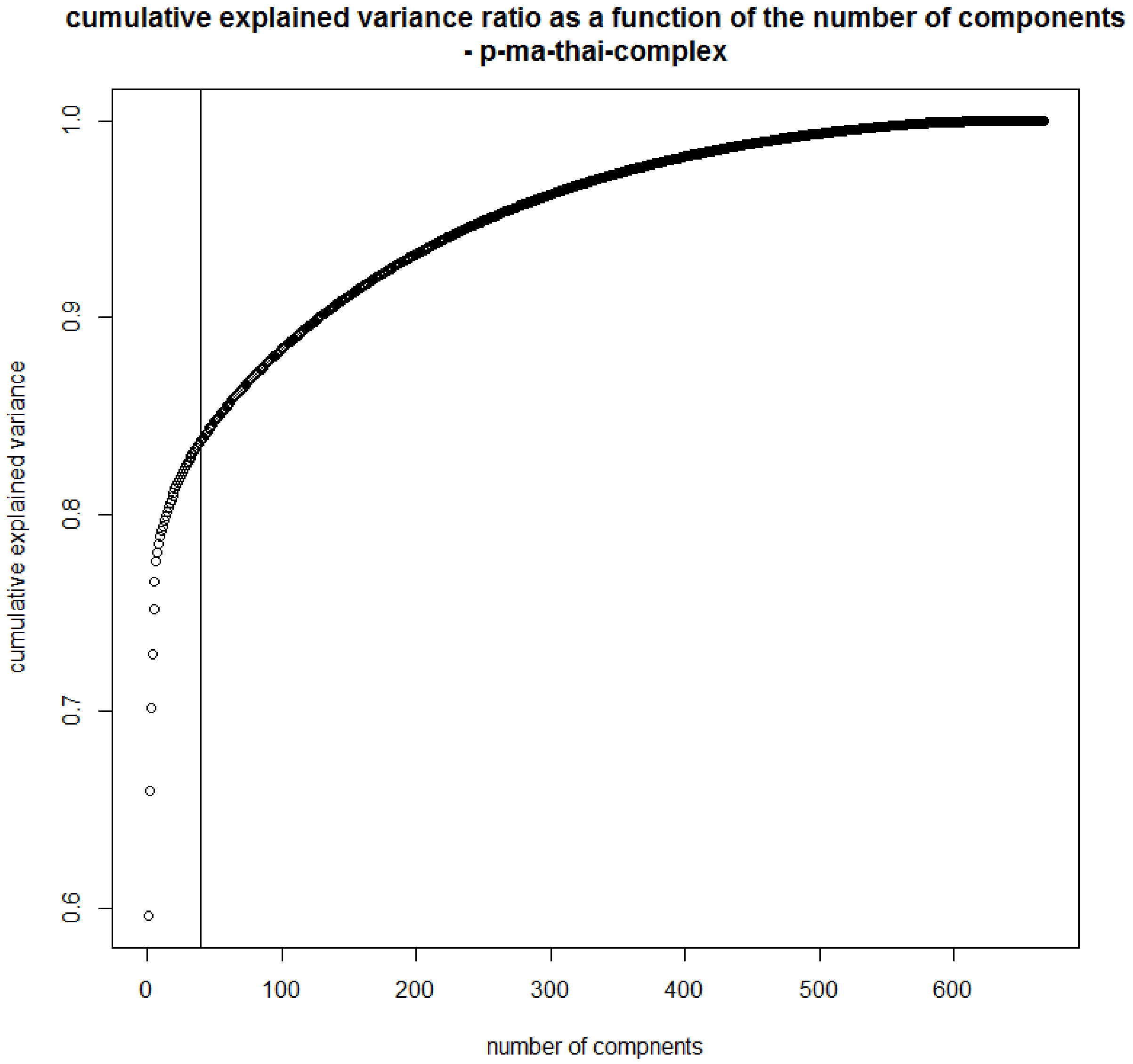

Fig. S4: The cumulative explained variance of the principal components (PCs) for the model 2.1. Model 2.1 gave the highest validation accuracy by introducing the first 40 PCs, indicated by the solid line.

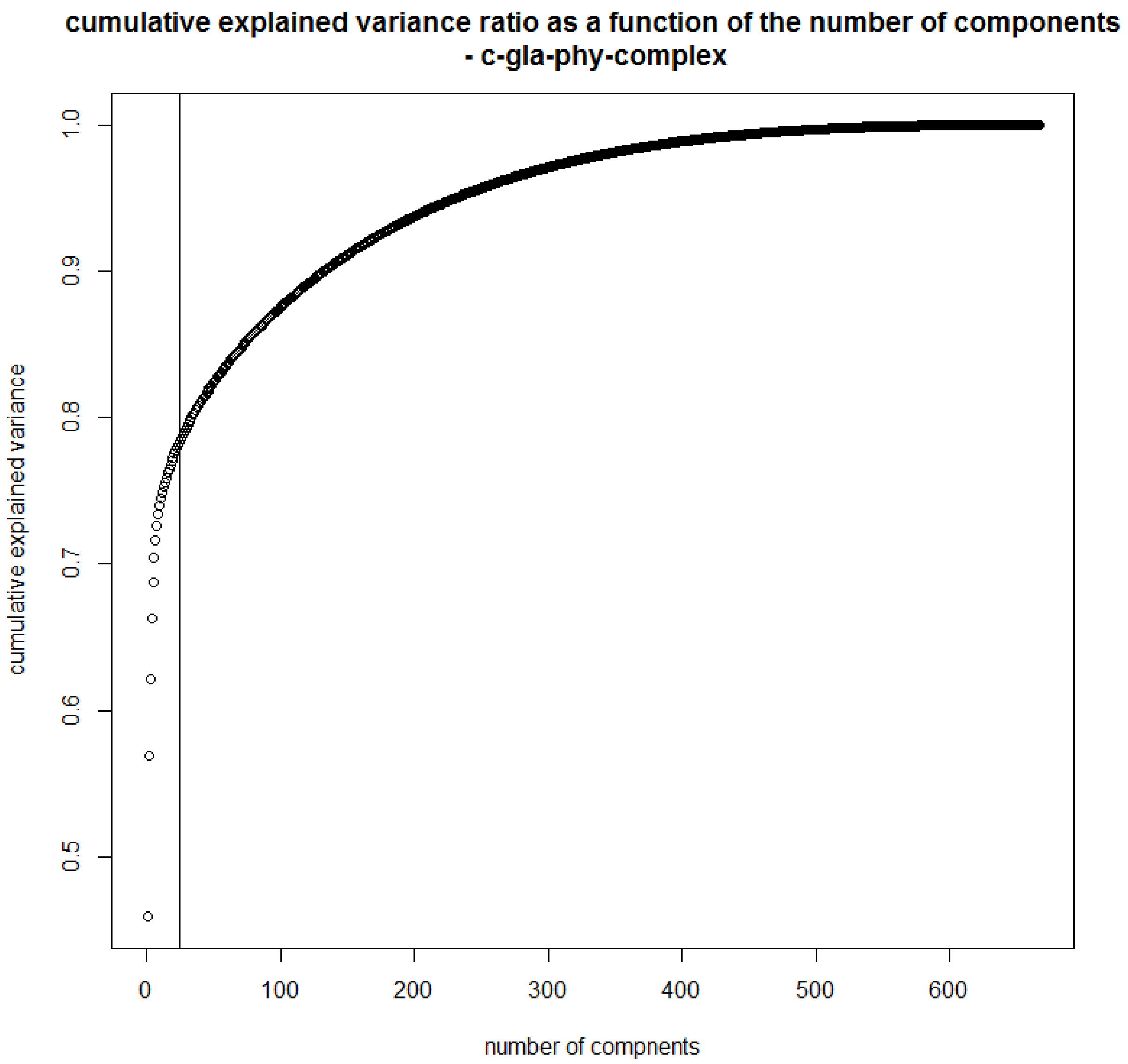

Fig. S5: The cumulative explained variance of the principal components (PCs) for the model 2.2. Model 2.2 gave the highest validation accuracy by introducing the first 25 PCs, indicated by the solid line.
